# Preventing spinal muscular atrophy through the national premarital screening program in Türkiye: an economic comparison with treatment costs

**DOI:** 10.55730/1300-0144.6169

**Published:** 2025-12-28

**Authors:** Gamze DUR, Gülcan TECİRLİ, Nurullah OKUMUŞ

**Affiliations:** 1 Department of Family Medicine, Faculty of Medicine, Afyonkarahisar Health Sciences University, Afyonkarahisar, Turkiye; 2 Health Services General Directorate, Ministry of Health, Ankara, Turkiye; 3 Deputy Minister, Ministry of Health, Ankara, Turkiye

**Keywords:** Spinal muscular atrophy, cost comparison analysis, genetic disease prevention, premarital carrier screening, in vitro fertilization with preimplantation genetic testing, public health policy

## Abstract

**Background/aim:**

Spinal muscular atrophy (SMA) is a severe neuromuscular disorder with high treatment costs and significant psychosocial burden. In 2021, Türkiye launched a national premarital SMA carrier screening program integrated with in vitro fertilization (IVF) with preimplantation genetic testing (PGT) services for couples identified as carriers. This study aimed to compare the costs associated with the carrier screening and prevention program versus a no-screening (treatment-only) scenario.

**Materials and methods:**

A cost comparison model was developed using data from the Turkish Statistical Institute, the Ministry of Health, and the Social Security Institution. The annual costs of SMA treatment (with nusinersen and risdiplam) and the costs associated with carrier screening, genetic counseling, and IVF with PGT were compared. Projections estimated 115 new SMA cases annually based on national birth rates and carrier frequencies.

**Results:**

The total annual cost of the premarital carrier screening and prevention program was estimated at TRY 112,801,201.6 (EUR 2,418,550.6). In contrast, the treatment of 115 new cases of SMA with nusinersen would cost TRY 1,091,623,700 in the first year alone, reaching a cumulative cost of TRY 2,183,247,377 over three years. The three-year cumulative cost for risdiplam treatment was calculated as TRY 1,196,414,795. The cost of preventing the birth of one SMA-affected child through the screening program was estimated as TRY 854,554.6, whereas treatment costs per child reached as high as TRY 18,984,759.6 with nusinersen.

**Conclusion:**

The SMA premarital carrier screening and prevention program in Türkiye significantly reduces healthcare expenditures and disease burden. Primary prevention through carrier screening is associated with lower overall costs than long-term treatment, offering both economic and social advantages for public health policy.

## 1. Introduction

Spinal muscular atrophy (SMA) is a group of inherited, progressive, neuromuscular diseases characterized by the degeneration of alpha motor neurons in the anterior horn of the spinal cord [1]. The condition is primarily caused by a deletion of the survival motor neuron 1 (SMN1) gene located on chromosome 5q13.2 [2–4]. It is one of the most common autosomal recessive disorders, with a worldwide incidence of 1 per 6000–10,000 live births [1–3]. The worldwide carrier frequency of SMA is about 1:51 [2,3], but it varies based on geographical origin, racial background, and ethnicity.

Although the incidence and prevalence of SMA in Türkiye remain unclear, the rates of autosomal recessive disorders such as SMA and thalassemia are expected to be elevated due to the continued prevalence of consanguineous marriages in certain regions. Hemoglobinopathy screening was initiated in 33 provinces in 2003 with the aim of reducing morbidity and mortality due to hemoglobinopathies, and was expanded nationwide in 2018 within the scope of the Hemoglobinopathy Screening Program (Evlilik Öncesi Hemoglobinopati Tarama Programı) carried out by the General Directorate of Public Health^1^. Through this initiative, significant epidemiological data on thalassemia have been collected, enabling reliable estimates of carrier frequency. Based on these data, the carrier frequency of SMA in Türkiye is presumed to be similar to that of thalassemia, though not higher.

Among SMA patients, the majority (approximately 92%–94%) have a homozygous deletion of the *SMN1* gene (solely exon 7 or exons 7 and 8) [2,5]. Another 2%–4% have a heterozygous deletion in *SMN1* along with a point mutation on the other allele [3,5], while the remaining 3%–4% have mutations in other genes, constituting non-5q SMA [5].

Five clinical phenotypes of 5q SMA have been recognized based on disease severity, age at onset, motor development, life expectancy, and functional abilities [1,2,6]. SMA Type 1 (Werdnig–Hoffmann disease) is the most common form, accounting for approximately 60% of cases, and is often fatal within the first two years of life due to respiratory complications [1,6]. SMA type 2 (Dubowitz’s disease) is the second most prevalent form (approximately 30%) and has a milder progression; affected individuals can sit unsupported but typically do not walk [1,6]. These phenotypes exist along a continuum rather than as distinct subtypes. For clinical and therapeutic decision-making, patients are often categorized functionally as nonsitters, sitters, or walkers [3]. In contrast to 5q SMA, non-5q forms are extremely rare and result from mutations in different genes. These include SMA with respiratory distress, Kennedy disease (spinobulbar muscular atrophy), and distal SMA. These forms may exhibit autosomal recessive, autosomal dominant, or X-linked recessive inheritance patterns [1].

Disease severity is strongly associated with both the age of symptom onset and the number of *SMN2* gene copies [1,7]. Although *SMN1* is the causal gene, *SMN2* acts as a major disease modifier, with higher copy numbers generally correlating with milder phenotypes [1–3,7]. This relationship has formed the basis for several targeted therapeutic developments.

Until recently, SMA management was limited to supportive care—physiotherapy, infection control, mechanical ventilation, and nutritional interventions—aimed at preventing complications such as respiratory failure, scoliosis, and joint contractures. However, the advent of biotechnological therapies has significantly altered the treatment landscape. While these therapies offer improved clinical outcomes, they also impose a substantial economic burden. Currently three pharmacologic agents have been approved by both the US Federal Drug Administration (FDA) and the European Medicines Agency (EMA) for SMA treatment. Nusinersen (Spinraza) is an antisense oligonucleotide that was first approved in 2016^2^ by the FDA [8]; onasemnogene abeparvovec-xioi (Zolgensma) is a gene replacement therapy [9]; and risdiplam (Evrysdi) is a small molecule modulator of *SMN2* splicing [10]. Research into additional therapies is ongoing, with early diagnosis recognized as critical to achieving optimal treatment efficacy.

Beyond pharmacotherapy, SMA requires a comprehensive multidisciplinary approach [11,12]. Patients often require long-term respiratory and nutritional support, with many dependent on mechanical ventilation or intensive care. The disease imposes significant psychological and financial strain on both their families and the broader healthcare system.

Preventive strategies such as preconception carrier screening, genetic counseling, and in vitro fertilization (IVF) with preimplantation genetic testing (PGT) offer an effective means of reducing the incidence of SMA and other autosomal recessive disorders. By interrupting the transmission of pathogenic genes to future generations, these interventions have the potential to significantly decrease disease burden over time.

Türkiye has a notable prevalence of rare diseases, necessitating a strong focus on identifying and implementing effective intervention strategies. In this regard, rare diseases, including SMA, represent a significant public health challenge. Recognizing the urgency of addressing these conditions, the Turkish Ministry of Health (MoH) has been actively engaged in developing comprehensive models and intervention programs aimed at reducing the burden of both rare and infectious diseases.

Nusinersen (Spinraza) was first approved for reimbursement in Türkiye for SMA Type 1 in July 2017, with coverage extended to Types 2 and 3 in February 2019 [13]. Most recently, risdiplam was included in the reimbursement list through an amendment to the Healthcare Implementation Communiqué (SUT), published in the Official Gazette on April 26, 2025 (Issue No. 32882), by the Social Security Institution of Türkiye (SSI) [14].

The aim of this study was to compare the costs of a national SMA prevention model with the treatment costs of individuals diagnosed with SMA. The model incorporated the National SMA Carrier Screening Program currently implemented in Türkiye, which includes premarital carrier screening, the provision of genetic counseling services for identified carrier couples, and IVF with PGT.

## 2. Materials and methods

This study adopted an analytical modelling approach that incorporated cost projections and statistical estimates to compare the expenses associated with SMA treatment and prevention. Data were sourced from official national agencies, including the SSI, Turkish Statistical Institute (TURKSTAT), and the MoH, along with affiliated institutions. The model compared treatment costs with those of a national SMA screening and prevention strategy, which included premarital carrier screening, genetic counseling, and IVF with PGT.

In Türkiye, couples planning to marry are required to undergo health screening at family health centers (FHCs), where hemoglobinopathy testing is routinely conducted. This infrastructure was considered suitable for integrating SMA screening alongside existing premarital testing within the Hemoglobinopathy Carrier Screening Program.

### 2.1. Screening algorithm

The premarital carrier screening algorithm developed by the General Directorate of Public Health^3^ can be summarized as follows:

- Individuals or couples applying to FHCs for premarital health certification or carrier screening are informed by the family physician about the screening process.
- Venous blood (2–3 cc) is drawn from each individual into an ethylenediaminetetraacetic acid tube, barcoded, and entered into the electronic health record system.
- A marriage certificate may be issued before the screening results are available. However, if screening is conducted preconceptionally, couples are advised to postpone pregnancy until the results are available.
- Blood samples are stored at 4 °C if not shipped the same day and sent to the Genetic Diseases Screening Laboratory (GDSL) via Provincial Health Directorate logistics.
- Samples are analyzed at the GDSL, and the results are electronically reported to the referring family physician as normal or suspicious.
- If both partners are found to be carriers, they are referred to a medical genetics specialist for confirmation and counseling.

Counseling outcomes and decisions are documented in the national e-health system (e-Nabız).

### 2.2. Screening method

The test method involves real-time polymerase chain reaction (RT-PCR) to detect copy number variations of exon 7 of the *SMN1* gene.

### 2.3. Cost analysis framework

Economic evaluation was conducted from a payer perspective, including only direct public expenditures related to screening, counseling, IVF with PGT, and pharmaceutical treatment. Patient out-of-pocket payment, hospitalization, and long-term care were excluded.

#### 2.3.1. Premarital carrier screening

To estimate screening costs, infrastructure expenditures, consumables, and personnel salaries were calculated based on tender data and prior research [15]. Between 2015 and 2024, approximately 562,000 couples got married annually in Türkiye^4^. Although the carrier frequency for SMA is not known with certainty, it has been reported to range between 1/50 and 1/40. In order to estimate the highest possible cost scenario, a carrier frequency of 1/40 was adopted in the present study. Within the national premarital screening program, individuals are initially screened at family health centers, and when one partner—operationally the male partner—is identified as an SMA carrier, the other partner is subsequently tested. Accordingly, it was estimated that approximately 14,050 individuals (562,000 × 1/40) would be identified as SMA carriers at the first stage of screening, resulting in an average of 576,050 individuals being tested annually.

#### 2.3.2. IVF with PGT for couples who are both SMA carriers

Based on the screening algorithm described above, the number of couples identified through the screening algorithm in which both partners were confirmed as SMA carriers was calculated as 351 (14,050 × 1/40). Given the high prevalence of consanguineous marriages in Türkiye (approximately 20%–25%), which has been shown to increase the frequency of autosomal recessive disorders by 30%–100%, a conservative 50% inflation factor was applied to this baseline estimate to reflect an upper-bound scenario. Accordingly, the annual number of couples identified as having both partners confirmed as SMA carriers was estimated at 527(357*1.5). The applied adjustment also accounts for regional heterogeneity, potential program losses, and false-negative test results reported in carrier screening programs. Data used to calculate the IVF with PGT costs were obtained from the SSI.

#### 2.3.3. Treatment of SMA

According to available data, it is estimated that 180–200 new cases of severe SMA are identified each year, with a total of approximately 3000–3300 SMA patients (including types 1–3) [16]. However, to obtain the lowest possible estimate in the cost calculations in the current study, and considering that the worldwide frequency of the disease is 1/6000–10,000, 1/10,000 was taken as the lowest frequency. Since an average of approximately 1,150,000 live births occurred annually in Türkiye between 2015 and 2024^5^, the calculations herein were based on 115 new cases per year (1,150,000 × 1/10,000). In the calculation of treatment costs, only medication costs were considered, and all other therapeutic procedures were excluded from the analysis.

## 3. Results

Results are given for the annual average cost of the premarital carrier screening for SMA, the cost of IVF with PGT for couples in which both partners are SMA carriers, the annual average cost of treatment for SMA with nusinersen and risdiplam, and the comparison between the treatment cost for SMA and the total cost of the SMA carrier screening program.

### 3.1. Annual average cost of the premarital carrier screening for SMA

According to the tender documents of the General Directorate of Public Health for the year 2024, a contract was signed with the company that placed the lowest bid for the SMA Carrier Screening Program PCR kit and all necessary consumables (including DNA extraction and related materials) on July 12, 2024, and an RT-PCR-based kit of 1,000,000 tests and all necessary consumables were purchased for a total of TRY 30,540,000.0^6^, indicating a unit cost of TRY 30.54 per test.

To obtain a more accurate cost estimate reflective of 2025 economic conditions, the unit cost per test was recalculated using the effective exchange rate published by the Central Bank of the Republic of Türkiye (CBRT; Türkiye Cumhuriyet Merkez Bankası) for 2025. Based on this updated rate, the unit cost of the test is projected to be TRY 39.65 [(30.54 / 35.9265) × 46.64]. Accordingly, the premarital screening of 576,050 individuals would result in a total consumable cost of TRY 22,840,382.5.

When calculating the required personnel for the carrier screening infrastructure, it is assumed that 576,050 tests will be performed annually over 220 working days, resulting in a daily workload of approximately 2618 tests. Given that each laboratory technician can process 100 tests per day, 26 laboratory personnel would be needed. In addition to these laboratory staff, support personnel (e.g., for reporting, archiving, and related administrative tasks) are also required, bringing the total personnel requirement to 40. Assuming an average gross monthly salary of TRY 50,000.0 (EUR 1072) per staff member, the estimated annual personnel expenditure amounts to approximately TRY 24,000,000.0 (EUR 514,579.8).

Capital equipment costs were assumed to be incurred for the infrastructure of the SMA screening program, and these costs should also be added to the total screening cost for the first year. The annual total cost of the devices required for the carrier screening infrastructure summarized in Table 1.

The total cost of premarital screening, including laboratory-related expenses, staff costs, and devices costs, is TRY 56,283,982.5 (EUR 1,206,774.9) (Table 2).

### 3.2. Cost of IVF with PGT for couples in which both partners are SMA carriers

In Article 63 of Social Insurance and General Health Insurance Law No. 5510, assisted reproductive treatment is listed among the health services to be financed and general conditions are specified. It is also explained in Article 2.4.4.I-Assisted Reproductive Treatment of the Health Implementation Communique (SUT)^7^. In addition, PGT for the birth of a healthy child is included in the scope of reimbursement. Accordingly, IVF with PGT may be required for a maximum of 527 couples per year (based on the above calculations); according to the SUT the cost of the IVF with PGT per patient was calculated as TRY 107,243.3 (Table 3).

The annual cost of IVF with PGT was calculated as (527 × TRY 107,243.3) TRY 56,517,219.1 (EUR 1,211,776.0).

### 3.3. Annual total cost of the SMA carrier screening program

When all the costs were summed up, the annual total cost of the SMA carrier screening program was calculated TRY 112,801,201.6 (EUR 2,418,550.6) (Table 4).

### 3.4. Annual average cost of treatment for SMA with nusinersen

According to the SMA Clinical Protocol^8^ published by the MoH, nusinersen is administered in four loading doses on days 0, 14, 28, and 63, followed by maintenance doses every 4 months. Based on this protocol, treatment continues as six doses in the first year and three doses in the following years. In this case, the cost of the treatment in terms of nusinersen in the first year will be (EUR 73,000 × 6) EUR 438,000. To reflect Social Security Institution (SSI) reimbursement practice, the official SSI exchange rate of EUR 1 = TRY 21.6721 was used. The cost per patient in the first year will be TRY 9,492,380 for SSI. For subsequent years, the cost can be calculated as (EUR 73,000 × 3) EUR 219,000.0 and TRY 4,746,189.9. However, the official cost data of nusinersen is not publicly available in Türkiye or in many other countries. For instance, a news report from China dated December 21, 2021, indicated that the price of nusinersen had been negotiated down from CNY 700,000 to CNY 33,000 over time^9^. Subsequently, in November 2022, nusinersen was granted marketing authorization in Türkiye under the license of Gen İlaç^10^. With risdiplam being added to the reimbursement list in 2025, it is anticipated that the price of nusinersen may have decreased further. In August 2020, the SSI updated the price of nusinersen to EUR 73,000 [17] and determined the Euro exchange rate for 2024 as TRY 21.6721 [18]. Cost calculations were conducted based on this price and exchange rate (Table 5).

Considering that there is a minimum annual average of 115 new cases, if no attempts are made to prevent the disease, the annual nusinersen treatment cost for these patients will be TRY 1,091,623,700.0 for the first year, and TRY 545,811,838.5 for the subsequent years.

### 3.5. Annual average cost of treatment for SMA with risdiplam

According to the Canadian Agency for Drugs and Technologies in Health, the annual per-patient cost of risdiplam was reported as CAD 93,456 for patients aged between 2 and 24 months, and between CAD 335,415 and 354,000 for those older than 24 months [19]. In a very recently published study, the annual cost per patient of nusinersen (Spinraza) was calculated as CAD 448,916 and the annual cost per patient of risdiplam (Evrysdi) was calculated as CAD 409,445, with a 12.5% discount of CAD 358,265.

Risdiplam has two forms, powder for oral solution [60 mg/bottle (0.75 mg/mL after reconstitution)] and tablet (5 mg)^11^. The daily dose for 2 years of age and older is <20 kg: 0.25 mg/kg ≥20 kg: 5 mg [18–20]. Moreover, the UK’s National Institute for Health and Care Excellence recently recommended risdiplam for reimbursement by the National Health Service for treating SMA in patients aged 2 months and older, provided that certain conditions are met, including a confidential discount on its list price^12^.

Assuming a newborn weighing 4 kg follows the 10th–15th percentile growth trajectory [21], it is estimated that the infant would require one bottle of risdiplam at a dosage of 0.15 mg/kg^13^ during the 0–2-month period. Between 3 and 24 months of age, the dosage increases to 0.20 mg/kg, requiring one bottle per month. For the period between 25 and 36 months, the dosage further increases to 0.25 mg/kg, necessitating two bottles per month. Once opened, a bottle of risdiplam has a shelf life of two months^14^.

Accordingly, the projected number of bottles required is 10 in the first year, 11 in the second year, and 20 in the third year. Due to the unavailability of an official list price for risdiplam, the market price of TRY 253,746.5^15^ per bottle was used (Table 6).

The cumulative per-patient cost of treatment with risdiplam over a 3-year period was determined as TRY 10,403,606.9 (EUR 223,061.9). For a cohort of 115 patients, the total cost was calculated as TRY 291,808,486.5 (EUR 6,256,614.2) in the first year, TRY 320,989,335.2 (EUR 6,882,275.6) in the second year, and TRY 583,616,973.0 (EUR 12,513,228.4) in the third year. Accordingly, the cumulative 3-year cost amounted to TRY 1,196,414,795.0 (EUR 25,652,118.2) (Table 7).

### 3.6. Comparison between the treatment (risdiplam and nusinersen) cost of SMA and the total cost of the SMA carrier screening program

**Figure 1:**
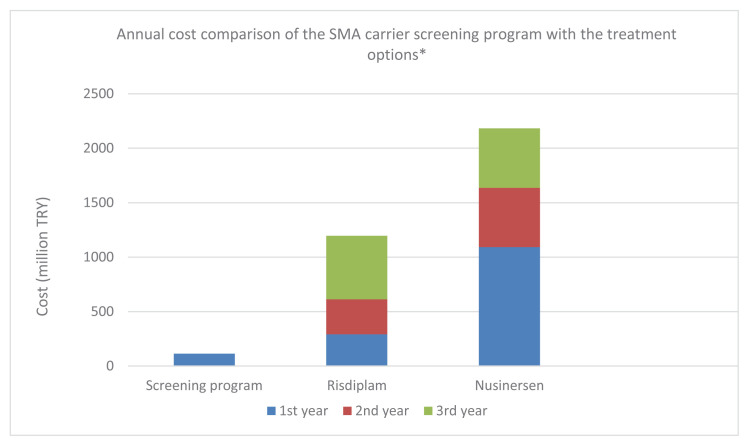
Annual cost of the SMA carrier screening and prevention program versus estimated 3-year cumulative treatment expenditures (risdiplam and nusinersen) for a cohort of 115 newly diagnosed SMA cases. *Decimal values reported in the table are rounded.

If the SMA screening and reproductive prevention program were not implemented, the estimated treatment expenditures for at least 115 newly diagnosed SMA cases per year would be substantial. When treated with risdiplam, the total cost for these patients would be TRY 291,808,486.5 in the first year, TRY 320,989,335.2 in the second year, and TRY 583,616,973.0 in the third year, resulting in a cumulative three-year cost of TRY 1,196,414,795.0. In the case of nusinersen treatment, the corresponding costs would be TRY 1,091,623,700.0 in the first year and TRY 545,811,838.5 in each of the second and third years, leading to a cumulative three-year cost of TRY 2,183,247,377.0 (Table 8, Figure 1).

**Figure 2:**
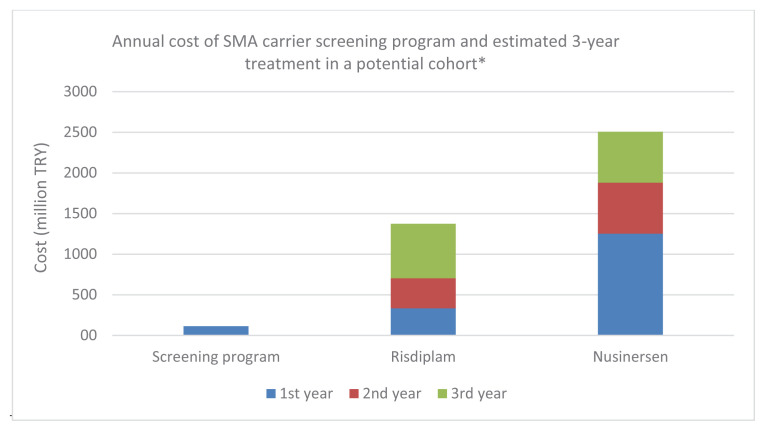
Annual cost of the SMA carrier screening program versus estimated 3-year cumulative treatment expenditures (nusinersen and risdiplam) for a cohort of 132 potential SMA cases. *Cost for the risdiplam and nusinersen is cumulative for the 2nd and 3rd year.

Within the framework of the SMA screening and reproductive prevention program, the annual number of married couples in which both partners are SMA carriers was estimated as 527. Given that each carrier couple has a 25% risk of having an SMA-affected child, the program has the potential to prevent the birth of approximately 132 SMA-affected children annually (527 × 0.25), assuming full uptake of IVF with PGT. While the total cost of the carrier screening program for the 132 potentially affected children remained constant at TRY 112,801,201.6, the cumulative treatment costs were estimated at TRY 1,373,276,112 for risdiplam and TRY 2,505,988,294 for nusinersen (Figure 2).

**Figure 3:**
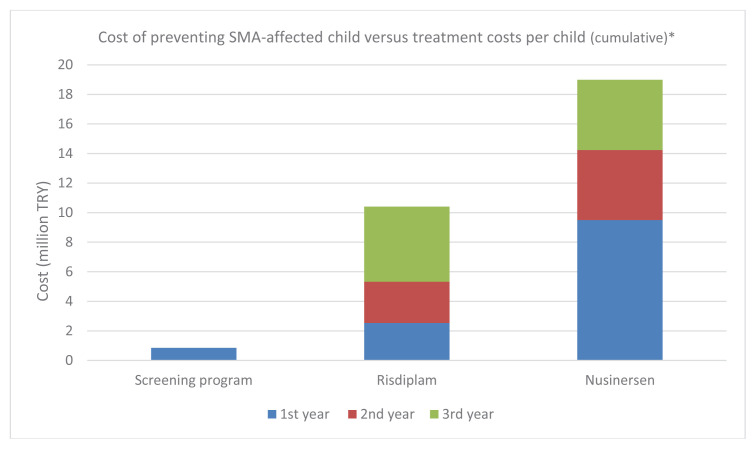
Cost per SMA case averted through the screening program versus estimated cumulative treatment expenditures (risdiplam and nusinersen) per patient. *Decimal values reported in the table are rounded.

Accordingly, the cost per SMA case averted was calculated by dividing the annual total program cost (screening plus IVF with PGT) by the estimated number of preventable cases. Based on this calculation, the cost required to prevent the birth of one SMA-affected child was estimated at TRY 854,554.6 (TRY 112,801,201.6/ 132). By contrast, assuming a minimum survival period of three years, the treatment cost per patient would amount to approximately TRY 10,403,606.9 for risdiplam and TRY 18,984,759.6 for nusinersen over the same period. These treatment costs would increase further with longer survival durations (Figure 3).

## 4. Discussion

Despite promising advances in therapeutic development, there are still no fully curative or side-effect-free treatment options for SMA. Moreover, existing therapies have significant limitations in terms of long-term efficacy, accessibility, and affordability [6,22]. Since 2008, the American College of Medical Genetics has recommended carrier screening for all couples, regardless of race or ethnicity [23]. Likewise, the American College of Obstetricians and Gynecologists advises offering screening to all women who are planning a pregnancy or are currently pregnant [24]. Both organizations emphasize that carrier screening and genetic counseling are ideally conducted before conception, enabling individuals to make informed reproductive choices.

In the current study, the national SMA Carrier Screening Program, comprising premarital carrier screening and IVF with PGT for carrier couples, resulted in markedly lower total costs, in addition to its societal benefits. According to the calculations conducted herein, the total cost of the screening program corresponded to only 10.3% of the first-year cost of nusinersen and 38.6% of the first-year cost of risdiplam. Even when applying a 10% increase to the total cost of the screening and IVF with PGT to account for unforeseen expenditures, the overall cost of the screening program still corresponded to only 11.4% of the first-year cost of nusinersen and 42.5% of the first-year cost of risdiplam. It is important to note that these figures include only the costs of the screening program and currently reimbursed medications. They exclude indirect and long-term costs such as the ongoing care of affected children, out-of-pocket expenses borne by families, societal productivity losses (e.g., absenteeism and presenteeism), and, perhaps most importantly, the emotional and psychological burden experienced by patients and their families. A similar program in Kuwait showed an 11% return on investment within five years, demonstrating its economic sustainability [25]. Although based on a different analytical framework, this finding provides supportive evidence on the economic sustainability of preventive SMA programs.

In contrast, the UK National Screening Committee (NSC) did not recommend SMA screening in 2018 due to uncertainties about test accuracy, unclear care pathways, and limited data on treatment efficacy in asymptomatic individuals. However, in 2023, the UK NSC initiated new cost-effectiveness modelling and an in-service evaluation, suggesting that screening recommendations may change with emerging evidence^16^. This evolving policy context underscores the importance of country-specific cost and programmatic evaluations, such as the present study.

Cost projections remained similar when using onasemnogene abeparvovec instead of nusinersen, due to the extremely high cost of this gene therapy. Although neither nusinersen nor onasemnogene abeparvovec has been found to be cost-effective for the treatment of infantile or later-onset SMA in many studies^17^ [26–28], nusinersen has been reimbursed in Türkiye since 2017. In parallel, two systematic reviews [29,30] emphasized persistent data gaps in SMA economic evaluations, including a lack of long-term efficacy and safety data. There remains a clear need for comprehensive, prospective studies encompassing both early-onset and later-onset SMA [31].

Genetic screening has its limitations. For example, carriers with two *SMN1* copies on the same chromosome (the 2+0 configuration) may have false-negative results; this genotype is estimated to occur in 3%–4% of the general population [1]. Additionally, de novo mutations account for approximately 2% of SMA cases due to unequal crossing over or gene conversion [1,2]. An individual with two or even three *SMN1* copies may still be a carrier, despite having normal gene dosage test results [1,3]. Furthermore, non-5q SMA cases cannot be detected via current screening methods [1]. Nevertheless, more than 90% of carriers are expected to be identified using current approaches [32,24,33].

For individuals with a family history of SMA, a more detailed genetic evaluation is recommended. For couples in which one partner is identified as a carrier, the other, regardless of their initial risk level, should also be tested. If both are carriers, referral to genetic counseling is essential [24]. One limitation of the current program is its inability to reach pregnancies outside the marital context. However, this limitation is likely to be less pronounced in the Turkish context. In countries with higher rates of non-marital births, alternative strategies, including preconception screening in primary care settings, may be more suitable. While not all unplanned pregnancies can be prevented, counseling for known carrier couples may reduce risk.

Even under optimal implementation conditions, the carrier screening program cannot eliminate all SMA cases. The outcomes of Türkiye’s Hemoglobinopathy Screening Program suggest that targeted genetic screening can significantly reduce the incidence of hereditary diseases. In provinces where premarital carrier screening is implemented, the number of babies born with hemoglobinopathies has dropped from over 300 per year to fewer than 100^18^. However, the absence of nationwide coverage has prevented the full eradication of thalassemia, despite over two decades of implementation. In contrast, countries with comprehensive and mandatory screening policies, such as Greece and the Turkish Republic of Northern Cyprus, achieved eradication within a shorter time frame [34].

Moreover, Andermann et al. [35] conceptualized genetic screening not merely as a test-based intervention, but as a multi-level public health strategy encompassing laboratory infrastructure, clinical services, and program management. According to this model, test validity and feasibility alone are insufficient; program success also depends on the inclusion of genetic counseling, follow-up care, quality control, and resource coordination. Policymaking in this area is a dynamic process that must evolve across the life cycle of the program, development, implementation, and evaluation, while also balancing the diverse values, expectations, and interests of multiple stakeholders. Genetic screening should therefore be approached not only as a technical procedure, but also as a complex socio-political and managerial intervention. These findings highlight the importance of national integration, strong policy commitment, and coordinated follow-up mechanisms for the success of the SMA screening program.

From an ethical standpoint, screening policies must balance clinical uncertainty, reproductive autonomy, disability perspectives, and cost-effectiveness. The availability of novel treatments adds complexity to parental expectations and decision-making. Screening and treatment should be evaluated holistically, within a patient-centered ethical framework.

Reproductive genetic carrier screening (RCS) enables individuals or couples to learn about their risk of passing on serious genetic conditions and is primarily aimed at supporting reproductive autonomy, especially before pregnancy. Programs such as Mackenzie’s Mission in Australia are designed to assess the ethical, economic, and practical aspects of RCS implementation. Ethical discussions have emphasized that the primary goal should be to inform reproductive decision-making rather than to reduce the incidence of genetic conditions. Couple-based result reporting is preferred for reasons of system efficiency and ethical responsibility. Future RCS programs should be designed to reflect community values and diversity, ensure informed consent, and promote equitable access to maximize their effectiveness and public acceptability [36].

Cultural context also shapes decision-making. A study from Saudi Arabia’s Jazan region found that many high-risk couples disregarded premarital counseling due to religious beliefs and different perceptions of disease risk. This underscores the need for culturally sensitive policy design [37].

From a disability ethics perspective, IVF with PGT raises concerns such as the expressivist objection, which argues that selecting against embryos with disabilities may devalue the lives of disabled people. Critics argue this can reinforce stigma and fail to embrace diversity. Some propose that gene editing, aimed at treatment rather than selection, may offer a more ethically acceptable path [38].

Attitudes among families affected by SMA also reflect this complexity. In a mixed-methods study, while 75% supported SMA screening, including preconception screening, motivations varied. Supporters emphasized reduced suffering and increased awareness; opponents raised concerns about social engineering, stigma, and the potential undervaluation of life with disability [39].

A structured health economics evaluation framework is critical for assessing the value of genetic screening programs, particularly for rare diseases such as SMA. In health economics analysis, commonly used methodologies include cost-effectiveness analysis, cost-utility analysis, cost-benefit analysis, and cost-consequence analysis, each offering different strengths depending on the policy question. These approaches are often supported by modeling techniques such as decision trees, Markov models, or microsimulations, tailored to the disease context and time horizon [40]. Given current data constraints and the policy focus of the present study, a cost comparison approach was considered the most appropriate first step.

Recent reviews in the literature suggest that genetic screening programs, especially those targeting conditions like hereditary breast and ovarian cancer or familial hypercholesterolemia, are generally found to be cost-effective when all relevant cost components are considered. These typically include the costs of testing and counseling, preventive interventions, and treatment, as well as indirect costs such as productivity losses. However, significant variability exists in model assumptions, such as willingness-to-pay thresholds, intervention designs, and population characteristics, which can limit the generalizability of results across different settings [40,41].

Türkiye’s Hemoglobinopathy Screening Program demonstrates that targeted premarital genetic screening can significantly reduce the incidence of hereditary diseases. Applying a similar approach to SMA could lead to a substantial reduction in new cases over time. This would result in significant long-term benefits by lowering treatment costs and reducing the burden on affected families.

In Türkiye, following legal and regulatory revisions, IVF with PGT treatment for carrier couples is reimbursed, and a national premarital carrier screening program was launched on December 27, 2021. Ideally, the best strategy is disease prevention. However, early treatment is also critical; presymptomatic diagnosis via newborn screening leads to significantly improved outcomes in SMA. Accordingly, SMA was added to the national newborn screening panel on May 9, 2022. In the coming years, real-world data will be needed to assess the long-term impact and effectiveness of this program.

In conclusion, primary prevention, which can only be achieved through preconception carrier screening, offers the greatest public health benefit in the context of SMA. Newborn screening functions as a secondary preventive measure, while even the timeliest treatment following diagnosis constitutes only tertiary prevention. Preventing disease onset from the beginning is not only the most effective medical approach, it is also the most socially and economically valuable. The psychosocial burden of SMA on individuals, families, and society far exceeds the direct medical costs. However, economic projections and comparative analyses strongly indicate that premarital carrier screening and IVF with PGT for at-risk couples can significantly reduce overall healthcare expenditures and societal burden. Though this model was developed for Türkiye, its applicability may extend to other countries facing similar challenges.

This study had several limitations. First, the cost analysis was projected on currently available unit prices and did not include indirect costs such as caregiver burden, productivity losses, or psychosocial impacts. Second, data on long-term health outcomes, especially for children born through IVF with PGT or detected via newborn screening, are limited. Third, ethical and cultural dimensions, while discussed, were not empirically measured within this analysis. Finally, the model assumes full adherence to program recommendations, which may not reflect real-world behavior. Future studies should aim to assess the long-term impact of the national SMA prevention program by incorporating comprehensive economic modeling, including lifetime cost estimates, indirect societal burdens, and health-related quality of life outcomes. Longitudinal follow-up using real-world data will be essential to evaluate program sustainability, uptake, and effectiveness over time. Additionally, future research should integrate perspectives from disability ethics and socio-cultural analysis to better understand the broader implications and public acceptance of preventive genetic programs.

## Figures and Tables

**Table 1:** Devices required for the carrier screening infrastructure.

Devices	Unit price	Number (min)	Cost (TRY)*
Automated DNA extraction and PCR setup system	EUR 250,000	2	23,320,000
High-Throughput Real-Time PCR System	USD 200,000	3	23,898,000
**Total cost**			47,218,000
**Annual cost** **			9,443,600

* 26.06.2025 effective exchange rate published by the CBRT (EUR 1 = TRY 46.64, USD 1 = TRY 39.83).

** To estimate the maximum cost, the depreciation period for the devices was determined as 5 years.

**Table 2:** Total annual cost of the premarital carrier screening.

Expenditure items	Cost (TRY)	Cost (EUR)
Laboratory supplies and related expenses	22,840,382.5	489,716.6
Required staff employment	24,000,000	514,579.8
Devices required for the carrier screening infrastructure (annually)	9,443,600	202,478.6
**Tota**l	56,283,982.5	1,206,774.9

**Table 3:** Cost of IVF with PGT per patient.

SUT code			Score	Price*	Level 3 hospital price**
	**1st cycle**				
P621043 (EK-2/C)		IVF	68,097.9	40,382.1	44,420.3
G101610 (EK-2/B)		Preimplantation genetic tests are used to help deliver healthy children.	63,254.6	37,510	41,260.9
704645 (EK-2/B)		Embryo freezing ***	1489.6	883.4	971.7
A03472 (EK-4/A)		Average cost of outpatient prescription drugs (Gonadotropin****)		4921.8	9843.6
	Subtotal				96,496.5
	**2nd cycle**				
P621046 (EK-2/C)		Freezing, embryo transfer after the procedure was performed	16,475.3	9769.8	10,746.8
	Subtotal				10,746.8
	**Total**				107,243.3

* Score multiplied by 0.593,

** price multiplied by 1.1,

*** paid once in their lifetime,

**** market price is TRY 8342,

SSI implies a 41% reduction from this price and multiplied by 2 since 2000 UI was assumed for each cycle, assuming that IVF with PGT was successful in the 2nd cycle.

**Table 4:** Total annual cost under the SMA carrier screening program.

	Cost (TRY)	Cost (EUR)
Screening cost	56,283,982.5	1,206,774.9
IVF with PGT cost	56,517,219.1	1,211,775.7
Total	112,801,201.6	2,418,550.6

**Table 5:** Annual cost of nusinersen at the official price.

	Official price (EUR) (a)	Number of doses administered per year (b)	Per patient cost per year (EUR) (c) (c = a × b)	Per patient cost (TRY)* (d) (d = c × 21.6721)	Total number of patients (e)	Total cost for all patients (TRY) (f) (f = d × e)	Total cost for all patients (EUR)** (g) (g = f / 46.64)	Cumulative cost for all patients (TRY)	Cumulative cost for all patients (EUR**)
1st year	73,000	6	438,000	9,492,380	115	1,091,623,700.0	23,405,310.4	1,091,623,700	23,405,310.9
2nd year	73,000	3	219,000	4,746,190	115	545,811,838.5	11,702,655.2	1,637,435,539	35,107,966.6
3rd year	73,000	3	219,000	4,746,190	115	545,811,838.5	11,702,655.2	2,183,247,377	46,810,621.3

* EUR 1 = TRY 21.6721 (SSI official EUR exchange rate),

** EUR 1 = TRY 46.64.

**Table 6:** Cost of risdiplam per patient.

	Number of bottles used per year (a)	Official price (TRY) (b)	Annual cost (TRY) (c) (c = a × b)	Annual cost (EUR) (d) (d = c / 46.64)	Cumulative cost for all patients (TRY)	Cumulative cost for all patients (EUR)
1st year	10	253,746.51	2,537,465.1	54,405.3	2,537,465.0	54,405.3
2nd year	11	253,746.51	2,791,211.6	59,845.9	5,328,676.7	114,251.2
3rd year	20	253,746.51	5,074,930	108,810.7	10,403,606.9	223,061.9

**Table 7:** Total cost of risdiplam for all patients.

	Annual cost per patient (TRY) (a)	Annual cost per patient (EUR) (b) (b = a / 46.64)	Total number of patients (c)	Total cost for all patients (TRY) (d) (d = a × c)	Total cost for all patients (EUR) (e = b × c)	Cumulative cost for all patients (TRY)	Cumulative cost for all patients (EUR)
1st year	2,537,465.1	54,405.3	115	291,808,486.5	6,256,614.2	291,808,486.5	6,256,614.2
2nd year	2,791,211.6	59,845.9	115	320,989,335.2	6,882,275.6	612,797,821.7	13,138,889.8
3rd year	5,074,930.2	108,810.7	115	583,616,973.0	12,513,228.4	1,196,414,795.0	25,652,118.2

**Table 8:** Cumulative cost of the SMA carrier screening and prevention program with the treatment options.

		Cumulative cost (TRY)	Cumulative cost (EUR)
**Screening and prevention program**	Annually	112,801,201.6	2,418,550.6
**Risdiplam**	1st year	291,808,486.5	6,256,614.2
	2nd year	612,797,821.7	13,138,889.8
	3rd year	1,196,414,795.0	25,652,118.2
**Nusinersen**	1st year	1,091,623,700.0	23,405,310.9
	2nd year	1,637,435,539.0	35,107,966.1
	3rd year	2,183,247,377.0	46,810,621.3

## Footnotes

1 General Directorate of Public Health (2023). Evlilik Öncesi Hemoglobinopati Tarama Programı [Online]. Website https://hsgm.saglik.gov.tr/tr/tarama-programlari/hemoglobinopati-kontrol-programi.html [accessed 10 October 2024]

2 FDA (2016). FDA approves first drug for spinal muscular atrophy [Online]. Website http://www.fda.gov/NewsEvents/Newsroom/PressAnnouncements/ucm534611.htm [accessed 10 March 2017]

3 General Directorate of Public Health (2025), Evlilik Öncesi Spinal Musküler Atrofi (SMA) Taşıyıcı Tarama Programı [Online], https://hsgm.saglik.gov.tr/tr/tarama-programlari/evlilik-oncesi-sma-tasiyici-tarama-programi.html#:~:text=Tarama%20testleri%20i%C3%A7in%20kan%20%C3%B6rnekleri,b%C3%BCnyesinde%20kurulan%20tarama%20laboratuvar%C4%B1na%20g%C3%B6nderilir. [accessed 30 June 2025]

4 TURKSTATS (2025), Demographic Statistics [Online]. Website https://data.tuik.gov.tr/Bulten/Index?p=Evlenme-ve-Bosanma-Istatistikleri-2024-54194 [accessed 19 June 2025]

5 TURKSTATS (2025), Birth Statistics [Online]. Website https://data.tuik.gov.tr/Bulten/Index?p=Dogum-Istatistikleri-2024-54196 [accessed 19 June 2025]

6 Public Procurement Agency, Electronic Public Procurement Platform (2024), SMA Carrier Screening Program PCR Kit and All Necessary Consumables (Including DNA Extraction and Consumables) Purchase [Online], Website https://ekapv2.kik.gov.tr/ekap/search/ihale-detay/9832257 [accessed 13 July 2025].

7 Social Security Institute (2025), Tüp Bebek Tedavisi [Online]. Website https://www.sgk.gov.tr/Content/Post/7de3f62d-ea0e-406f-871a-8644f02a7e3c/Tup-Bebek-Tedavisi-2026-01-09-04-09-54 [accessed 30 November 2025]

8 Ministry of Health (2025), SMA Clinical Protocol. Website https://shgmargestddb.saglik.gov.tr/TR-89971/spinal-muskuler-atrofi-sma-klinik-protokolu.html [accessed 30 June 2025]

9 China Daily (2021), China increases accessibility to rare disease treatment. Website https://www.chinadaily.com.cn/a/202112/21/WS61c1831da310cdd39bc7cad0.html [accessed 2 July 2025]

10 Public Disclosure Platform (Kamuyu Aydınlatma Platformu) (30 November 2025). Website https://www.kap.org.tr/tr/Bildirim/1083111 [accessed 30 June 2025]

11 Medscape (2025), Risdiplam (Rx), Website https://reference.medscape.com/drug/evrysdi-risdiplam-4000042#0 [accessed 13 July 2025]

12 WHO (2024), Proposal for the addition of risdiplam to the who model list of essential medicines for the treatment of children and adults with spinal muscular atrophy. Website: https://cdn.who.int/media/docs/default-source/2025-eml-expert-committee/addition-of-new-medicines/a.24_risdiplam.pdf?sfvrsn=a0920ef9_9 [accessed 13 July 2025]

13 Mayo Clinic (2025), Risdiplam (oral route), Website https://www.mayoclinic.org/drugs-supplements/risdiplam-oral-route/description/drg-20492099 [accessed 2 July 2025]

14 Medscape (2025), Risdiplam (Rx), Website https://reference.medscape.com/drug/evrysdi-risdiplam-4000042#11 [accessed 2 July 2025]

15 Türk İlaç Rehberi (2025), EVRYSDI 0.75 mg/ml oral çözelti. Website https://www.ilacrehberi.com/ilac-fiyatlari/8699505593210/ [accessed 13 July 2025]

16 UK NSC screening recommendation, Antenatal and newborn screening programme, Spinal muscular atrophy, https://view-health-screening-recommendations.service.gov.uk/sma/

17 National Centre for Pharmacoeconomics (2017). Cost-effectiveness of nusinersen (Spinraza) for the treatment of 5q spinal muscular atrophy (SMA). Website http://www.ncpe.ie/wp-content/uploads/2017/08/Summary-Nusinersen.pdf [accessed 12 Jan 2018].

18 Republic of Türkiye Ministry of Health-General Directorate of Public Health (Department of Child and Adolescent Health), 2018, Premarital Hemoglobinopathy Screening Program Field Guide.https://hsgm.saglik.gov.tr/tr/tarama-programlari/hemoglobinopati-kontrol-programi.html [accessed 29 January 2026]
